# 射频消融术联合化疗治疗进展期非小细胞肺癌远期疗效的回顾性研究

**DOI:** 10.3779/j.issn.1009-3419.2017.10.03

**Published:** 2017-10-20

**Authors:** 淑卉 杜, 达 秦, 睿奇 庞, 叶青 张, 思琪 赵, 牧 胡, 修益 支

**Affiliations:** 100053 北京，首都医科大学宣武医院胸外科 Department of Toracic Surgery, Xuanwu Hospital, Beijing 100053, China

**Keywords:** 肺肿瘤, 射频消融术, 化疗, 远期疗效, Lung neoplasms, Radiofrequency ablation, Chemotherapy, Long-term efcacy

## Abstract

**背景与目的:**

肺癌射频消融术（radiofrequency ablation, RFA）联合化疗对于晚期非小细胞肺癌（non-small cell lung cancer, NSCLC）近期疗效的改善具有一定的意义，但其是否可以改善晚期NSCLC患者的远期生存仍然存在争议。本研究回顾性分析了射频消融术联合化疗治疗与单纯化疗对于晚期NSCLC患者的远期疗效的差异。

**方法:**

选择2012年9月-2015年12月在首都医科大学宣武医院胸外科进行肺癌射频消融术并进行化疗的Ⅲb期与Ⅳ期NSCLC患者77例作为实验组，选择同期只进行化疗未进行射频消融术的Ⅲb期与Ⅳ期NSCLC患者56例作为对照组。对两组患者进行电话随访，询问患者目前的生存情况与死亡时间。运用R3.4.1软件进行统计分析，使用“survival”程序包，用卡方检验比较两组数据基线水平，运用*Cox*比例风险模型处理混杂偏倚，并使用协变量均值取代法绘制经过校对的生存曲线。

**结果:**

两组基线齐，实验组的1年生存率为70.74%，2年生存率为39.31%，中位生存期为22.1个月；对照组的1年生存率为54.54%，2年生存率为19.49%，中位生存期为18.1个月。组间差异有统计学意义（*P* < 0.05）。

**结论:**

对于Ⅲb期-Ⅳ期NSCLC患者，肺癌射频消融术联合化疗治疗可以显著提高患者的2年生存率。

## Abstract (en)

**Background and objective:**

Radiofrequency ablation (RFA) combined with chemotherapy has a certain short-term therapeutic effect for the treatment of advanced non-small cell lung cancer (NSCLC), but whether it can improve the long-term survival rate of patients is still controversy. This study retrospectively analyzed the difference of long-term efficacy between RFA combined with chemotherapy and chemotherapy alone in the treatment of patients with advanced NSCLC.

**Methods:**

A total of 77 patients with stage Ⅲb and stage Ⅳ NSCLC who underwent radiofrequency ablation and chemotherapy in the Department of Thoracic Surgery, Xuanwu Hospital, Capital University of Medical Sciences from September 2009 to December 2015 were enrolled as the treatment group. Chemotherapy with no radiofrequency ablation was performed in 56 patients with stage Ⅲb and stage Ⅳ NSCLC as the control group. Two groups of patients were followed up by telephone about their living conditions. "Survival" package of R software version 3.4.1 was used for statistical analysis. Two sets of data baseline levels were tested by chi-square test. The bias was processed by *Cox* regression model and the survival curve was plotted using covariate mean substitution method.

**Results:**

The first-year survival rate of the treatment group was 70.74%, the two-year survival rate was 39.31% and the median survival time was 22.1 months. The one-year survival rate was 54.54% in the control group, the two-year survival rate was 19.49%, the median survival for 18.1 months. The long-term survival rate of the treatment group was better than that of the control group (*P* < 0.05, OR=0.571).

**Conclusion:**

Radiofrequency ablation of lung cancer combined with chemotherapy can significantly improve the 2-year survival rate of patients with stage Ⅲb and stage Ⅳ NSCLC.

经流行病学统计^[1, 2]^，随着工业化的发展和生活习惯的改变，肺癌已替代肝癌成为我国首位恶性肿瘤死亡原因，占全部恶性肿瘤死亡的22.7%，其中非小细胞肺癌（non-small cell lung cancer, NSCLC）又占其大多数。随着我国人口老龄化、城镇现代化和农村城市化进程的加快中环境的影响与早期NSCLC症状的不典型，70%-80%的患者就诊时已至晚期，丧失了手术根治的指征^[3]^。此时，化疗成为大多数患者的选择，但是由于患者对化疗的耐受性差，这种治疗不仅为患者造成了痛苦，疗效也不尽如人意。近年来，肺癌RFA作为一种局部治疗方法^[4]^，逐渐应用于NSCLC的治疗。射频消融术（radiofrequency ablation, RFA）具有微创，效果确切，并发症少，患者耐受良的特点，对于Ⅲb期-Ⅳ期NSCLC患者起到了一定的临床疗效^[5]^。RFA联合化疗成为了治疗晚期NSCLC的新思路。很多研究^[6-8]^表明，RFA联合化疗对于晚期NSCLC近期疗效的改善具有一定的意义，但是，RFA联合化疗是否可以改善晚期NSCLC患者的远期生存仍然存在争议^[8, 9]^。本文将就RFA联合化疗对晚期NSCLC患者远期疗效展开回顾性研究。

## 1 资料与方法

### 1.1 研究对象

#### 1.1.1 研究对象的临床资料

实验组入组条件：①患者为首诊，或者为入院前经过初步诊断但未进行任何实质性治疗的患者。就诊时间为2012年9月-2015年12月。②就诊地点为首都医科大学宣武医院胸外科。③患者在住院期间进行了肺癌射频消融术治疗。④在射频消融术治疗的同时，患者进行了同期的全身化疗。⑤患者的肿瘤分期为Ⅲb期或Ⅳ期，肿瘤分期标准为国际抗癌联盟（Union for International Cancer Control, UICC）第七版肺癌TNM分期标准。⑥患者病理分型为NSCLC患者。⑦*EGFR*基因检测为阴性，或者*EGFR*基因检测为阳性但未接受靶向药物治疗，或者接受靶向药物治疗后不耐受或耐药即停药的患者。

排除条件：①存在严重的化疗药物不耐受，不能进行化疗。②患者的体能状态（performance status, PS）评分 > 2分。③患者的预期生存 < 6个月。④患者有已知重大的活动性感染。⑤具有严重的无法纠正的酸碱代谢紊乱以及水电解质失衡。⑥患者心功能分级为Ⅲ级或Ⅲ级以上。⑦患者患有高血压，分型在高危型或极高危型。⑧患者有心脑血管疾病记录史，包括冠心病、脑梗死、脑出血等疾病。⑨患者存在明确诊断的肝肾功能不全。⑩有免疫缺陷病史，具有先天或后天的免疫缺陷病，或者近期进行器官移植，长期服用免疫抑制剂或糖皮质激素类药物。患者进行除了化疗与肺癌射频消融术以外的治疗，如其他减瘤手术或服用靶向药物等。

对照组入组条件：①患者为首诊，或者为入院前经过初步诊断但未进行任何实质性治疗的患者。就诊时间为2012年9月-2015年12月。②就诊地点为首都医科大学宣武医院胸外科。③患者进行了全身化疗。④患者的肿瘤分期为Ⅲb期或Ⅳ期，肿瘤分期标准为UICC第七版肺癌TNM分期标准。⑤患者病理分型为NSCLC患者。⑥*EGFR*基因检测为阴性，或者*EGFR*基因检测为阳性但未接受靶向药物治疗，或者接受靶向药物治疗后不耐受或耐药即停药的患者。

排除条件：①存在严重的化疗药物不耐受，不能进行化疗。②患者的PS评分 > 2分。③患者的预期生存 < 6个月。④患者有已知重大的活动性感染。⑤具有严重的无法纠正的酸碱代谢紊乱，以及水电解质失衡。⑥患者心功能分级为Ⅲ级或Ⅲ级以上。⑦患者患有高血压，分型在高危型或极高危型。⑧患者有心脑血管疾病记录史，包括冠心病、脑梗死、脑出血等疾病。⑨患者存在明确诊断的肝肾功能不全。⑩有免疫缺陷病史，具有先天或后天的免疫缺陷病，或者近期进行器官移植，长期服用免疫抑制剂或糖皮质激素类药物。患者进行除了化疗以外的治疗，如任何减瘤手术或服用靶向药物等。

共纳入实验组患者77例，对照组患者56例。

#### 1.1.2 研究对象的随访

对实验组与对照组的所有患者进行电话随访。其中，实验组随访时间平均为31个月。对照组随访时间平均为35个月。经过两独立样本的*t*检验，*t*=-1.661，*P*=0.1（*P* > 0.05），两组随访时间无统计学差异。对电话随访员进行培训，统一随访标准。记录每例患者的生存状态，使用美国东部肿瘤协作组（Eastern Cooperative Oncology Group, ECOG）评分标准评价患者的PS，如果患者死亡记录患者的死亡时间，具体到月，并且记录患者是否为肿瘤相关性死亡。

### 1.2 射频消融术的操作方法

根据肿瘤的部位让患者选择合适的体位，常规计算机断层扫描（computed tomography, CT）平扫并行三维图像重组，在预定体表位置放置栅形定位器行定位扫描。CT扫描病灶确定最大层面，用定位灯作皮肤标记，用光标测进针方向、角度和深度。患者皮肤常规消毒铺巾，以2%利多卡因10 mL局部麻醉并保留注射器于胸壁上，重复局部CT扫描确定进针位置和进针角度，在患者平静呼吸下使用18 G伞状美国RITA射频消融针经标记进针位置进针至靠近病灶的位置。重复局部CT扫描确认针尖达到肿瘤内部位置满意，本研究中进针长度平均为（6.95±1.77）cm。根据肿瘤的大小选择打开针伞的长度，本研究打开针伞的长度平均为（3.81±1.04）cm，保证消融灶边缘超过病灶边缘0.5 cm-1 cm，以杀死肿瘤生长最活跃的周边部分。重复CT扫描确认消融范围无误后连接射频消融主机，开始射频消融，本研究设定温度为（90.1±1.71）℃，消融时间根据肿瘤直径的大小而定，平均为（25.32±8.16）min，并进行多点温度测定。

### 1.3 统计方法

#### 1.3.1 统计描述

统计了实验组的77例患者进行射频消融术时所消融的病灶数目，对于远处转移灶的射频消融处理情况，以及在术中及术后中出现的与肺癌射频消融术直接相关的并发症，并统计各并发症出现的比例。

#### 1.3.2 单因素分析

数据预处理后载入R3.4.1软件进行统计分析，使用χ^2^检验比较实验组和对照组性别、肿瘤位置、肿瘤的个数、肿瘤的病理类型、肿瘤分期、化疗方案水平的差异。使用独立样本的*t*检验来对比两组间患者年龄，肿瘤大小与患者化疗次数水平的差异。

#### 1.3.3 多因素分析

运用R3.4.1软件进行统计分析，使用“survival”程序包，使用*Cox*比例风险模型，使用进入法，将实验组和对照组的组别、年龄、性别、肿瘤位置、肿瘤最大直径、肿瘤的病理类型、肿瘤分期、化疗方案与化疗周期数纳入统计。并使用协变量均值取代法绘制经过校对的生存曲线。

## 2 结果

### 2.1 统计描述

经过统计，在实验组的77例患者中，66例患者进行了1个病灶的射频消融术处理，10例患者进行了2个病灶的射频消融术处理，1例患者进行了3个病灶的射频消融术处理，消融病灶数目共计为89个。从患者的临床资料中未发现远处转移灶的射频消融术处理。

另外，术中及术后患者出现了发热、咳嗽、咳痰、咯血、胸痛、胃肠道反应的不良反应，发生的例数以及所占的比例如表 1所示。

**1:** 射频消融术后不良反应情况 Side effects after RFA

	Fever	Cough	Expectoration	Hemoptysis	Chest pain	Gastrointestinal reaction	Total
Number	13	2	3	2	8	2	30
Proportion	16.9%	2.6%	3.9%	2.6%	10.4%	2.6%	39.0%
RFA: radiofrequency ablation.							

### 2.2 单因素分析

实验组与对照组的基线数据对比如下。其中两组间性别、肿瘤位置、肿瘤的个数、肿瘤的病理类型、肿瘤分期、化疗方案水平差异的对比如表 2。两组患者年龄、肿瘤大小、患者化疗次数以及化疗剂量水平的差异的对比如表 3。从表中可得，两组在基线上的对比，每个水平上*P* > 0.05，基线齐，具有可比性，控制了选择性偏倚。

**2:** 实验组与对照组的基线数据比较 Baseline data comparison between the treatment group and the control group

	Treatment group (*n*=77)	Control group (*n*=56)	*χ*^2^	*P*
Gender			1.122	0.290
Male	47	40		
Female	30	16		
Location				
Left	39	24	0.508	0.476
Right	38	32		
Upper	43	32	0.305	0.859
Middle	4	4		
Lower	30	20		
Tumor number			1.340	0.247
Single	42	37		
Multiple	35	19		
Pathology			3.742	0.053
Adenocarcinoma	55	30		
Squamous carcinoma	22	26		
Staging			1.870	0.172
Stage Ⅲb	12	15		
Stage Ⅳ	65	41		
Chemotherapy regimen			6.227	0.400
NP	1	1		
TP	9	11		
GP	36	32		
DP	9	3		
AP	12	6		
Single-agent gemcitabine	5	1		
Single-agent pemetrexed	5	2		
NP: Vinorelbine+Cis-platinum; TP: Paclitaxel+Cis-platinum; GP: Gemcitabine+Cis-platinum; DP: Docetaxel+Cis-platinum; AP: Pemetrexed+Cis-platinum.				

**3:** 实验组与对照组的基线数据比较 Baseline data comparison between the treatment group and the control group

	Mean of treatment group	Mean of control group	*t*	*P*	95%CI
Age (yr)	61.8	62.2	0.258	0.797	-2.778-3.613
Tumor diameter (cm)	4.2	3.7	-1.422	0.158	-1.117-0.183
Number of chemotherapy	4.2	4.5	0.611	0.543	-0.579-1.092
Chemotherapy dose					
TP					
Paclitaxel (mg)	213.3	247.5	0.916	0.388	-52.872-121.205
Cis-platinum (mg)	341.7	242.5	-1.118	0.289	-296.078-97.745
GP					
Gemcitabine (mg)	1, 840.0	1, 878.6	0.556	0.581	-0.101-0.178
Cis-platinum (mg)	286.8	292.5	0.118	0.907	-92.596-104.096
DP					
Docetaxel (mg)	110.0	120.0	1.000	0.500	-117.062-137.062
Cis-platinum (mg)	275.0	245.0	-0.099	0.931	-1, 350.534-1, 290.534
AP					
Pemetrexed (mg)	741.7	795.0	1.612	0.165	-30.582-137.248
Cis-platinum (mg)	285.0	148.0	-1.714	0.125	-321.535-47.535
Single-agent pemetrexed					
Pemetrexed (mg)	633.3	750.0	0.819	0.483	-391.921-625.254

### 2.3 多因素分析

运用R3.4.1软件进行统计分析，使用“survival”程序包，使用*Cox*比例风险模型，将实验组和对照组的组别、年龄、性别、肿瘤位置、肿瘤最大直径、肿瘤的病理类型、肿瘤分期、化疗方案与化疗周期数纳入统计。

处理混杂偏倚以后，结果由表 4可见，其中，实验组的1年生存率为70.74%，2年生存率为39.31%，中位生存期为22.1个月；对照组的1年生存率为54.54%，2年生存率为19.49%，中位生存期为18.1个月。其中，患者的死亡原因均为肿瘤相关性死亡。组别对患者的生存时间具有显著的统计学差异（*P* < 0.05），OR=0.571，说明化疗联合射频消融术治疗患者的远期疗效高于只进行化疗的患者具有统计学差异。使用协变量均值取代法绘制校正了混杂因素的生存曲线，如图 1。

**4:** 实验组与对照组的多因素*Cox*回归模型各因素的描述 Description of the factors in the multivariate *Cox* regression model of the treatment and control groups

	B	SEM	*P*	OR	95%CI
Group	-0.560	0.281	0.046	0.571	0.991-0.329
Gender	0.661	0.304	0.030	1.936	3.514-1.067
Diameter of tumors	0.429	0.183	0.019	1.536	2.200-1.072
Location	-0.547	0.238	0.021	0.578	0.363-0.924
Pathology	-1.575	0.691	0.023	0.207	0.053-0.801
Staging	1.503	0.416	0.000	4.497	1.991-10.161

**1:**
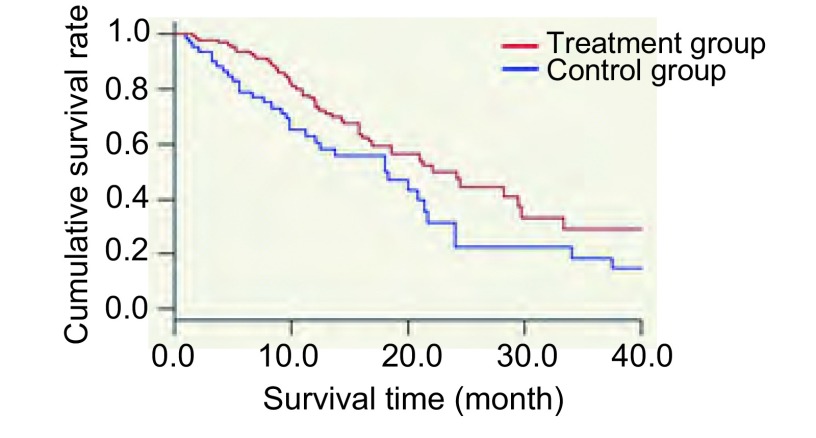
经过校正混杂因素的生存曲线 Survival curve of corrected confounding factors

## 3 讨论

自从Dupuy等^[4]^在2000年首次将射频消融术引入到肺癌的治疗以来，很多前瞻性研究^[6-8]^表明，肺癌射频消融术对于晚期肺癌患者具有良好的近期疗效。但对于其是否可以改善患者的远期生存率，一些研究^[8, 9]^持有矛盾的意见。射频消融术作为一种减瘤手术，如果其治疗效果只局限于近期疗效而不能改变患者预后，其临床意义将会很有限。于是，本研究选择了基线可比，一般状况良好的两组患者来探究肺癌射频消融术的远期疗效，得到了化疗联合射频消融术治疗患者的远期疗效高于只进行化疗的患者的结论。下面是针对本研究几个问题的讨论。

对于肺癌射频消融术联合化疗组的远期生存率高于单纯化疗组的原因，本研究有以下观点供探讨。

首先，从肺癌射频消融术的原理分析。肺癌射频消融术本质是通过射频消融仪发出的频率为460 HZ-500 HZ，功率超过200 W的高频电流，通过展开为伞状，均匀分布于肿瘤内部的细电极针，发出射频波，使肿瘤内部的分子相互摩擦，产生90 ℃-120 ℃的高热^[3]^。而在60 ℃-100 ℃的温度下，蛋白质就会发生凝固性坏死^[10]^。这样，肿瘤细胞会产生不可逆的损伤，达到肿瘤患者减轻肿瘤负荷的作用。这种局部减瘤的效果，可能是提高患者远期生存率的一个原因。

其次，肺癌射频消融术后，在消融灶的周围会形成一层凝固带，这层凝固带可以阻止周围血管组织对肿瘤细胞的供血，这种供血途径的阻断一方面可以阻止肿瘤获得营养物质，阻止肿瘤进一步发展，更重要的一方面，这层凝固带可以阻止肿瘤向远处转移，防止肿瘤复发，复发率的减少可能是患者远期生存率提高的一个原因。

第三，正常肺组织由肺泡组织构成，肺泡组织中空气占很大的比重，热量在空气中不易传导，另外肺内血液供应充足，大小血管众多，血液的流动也会带走一部分热量。而肿瘤属于实性结构，进行肺癌射频消融术治疗时，热量在肿瘤组织内充分保留，而在肿瘤外的肺组织内流失很快^[11]^。这将使手术效果最大化。事实上，有研究^[12]^论证，肺组织中的癌肿是全身各组织中最适合射频消融术治疗的一个种类。这种优秀的手术效果可能是患者得到远期生存的一个原因。

第四，有研究^[13]^认为，肺癌射频消融术后坏死的肿瘤组织可以作为原位抗原诱发持续的T细胞活化，对全身进行免疫激活，进而产生对于NSCLC细胞长期的特异性免疫反应。也有学者^[14]^认为，射频的热效应会增强机体的非特异性免疫反应。总之，无论是特异性还是非特异性免疫反应，射频消融术后机体对于NSCLC细胞的免疫力得到了提升^[15]^，这可能是患者远期生存的一个原因。

最后，有学者^[14]^认为，富氧细胞对化疗的敏感性强于乏氧细胞，而射频消融术对于乏氧细胞的敏感性强于富氧细胞。射频消融术针对的是影像学上可见的大的肿瘤病灶，而化疗是一种全身治疗，可以治疗亚临床病灶与小病灶，两种治疗方法可以互相补充。另外，高温刺激可能会有增强化疗药物的细胞毒性，逆转化疗药物的耐药性等作用。但这些猜想还缺乏确切的针对肺癌的研究来证实其可靠性。

经过统计，本研究发现在进行过肺癌射频消融术治疗的77例患者中，其中有13例出现了发热，8例出现胸痛，2例出现咯血，7例出现咳嗽、咳痰及胃肠道反应。共计有30例出现不良反应，占总数的39.0%，有接近61.0%的患者术后一般情况好，未出现明显的不良反应。

有文献^[16]^报道，肺癌射频消融术后的不良反应有发热、胸痛、气胸等轻微并发症，以及出血、咯血、肺炎和空洞等严重并发症。其中，有报道^[17]^称轻微并发症的发生率在21.3%-64.9%之间，严重并发症的发生率在3%-24.5%之间。本研究并发症的发生率合乎其统计的发生率。其中，本研究中多发的并发症为发热与胸痛，下面将重点探讨这两者的发生机制及处理。

本研究中发热的发生率为16.9%，是发病率最高的并发症。一般认为^[18]^，术后发热分为两种：第一种作为消融后综合征^[19]^的一个症状，常与恶心、乏力等一般症状伴发。主要是由于肿瘤结节消融后坏死组织的吸收以及机体对于射频消融热刺激的反应引起。此种发热一般 < 38.5 ℃，少数超过39 ℃，持续3 d-5 d，具有自限性，不需要特殊处理；第二种发热是在继发感染后的发热，如果存在继发于感染的发热，即应积极抗感染治疗。

本研究中胸痛的发生率为10.4%，仅次于发热。胸痛的原因可能为胸膜神经受到射频消融针的高温损伤所致。疼痛程度不重，患者一般可以耐受，如果无法耐受可以给予解热镇痛抗炎药治疗，不影响患者预后。但是对于原有基础心血管疾病的患者，要注意与射频消融术后诱发的心源性胸痛鉴别。

综上所述，肺癌射频消融术后严重并发症的发病率低。即使发生了并发症，程度轻，具有成熟的应对方法。可以说，肺癌射频消融术是一种相对安全的治疗方法，值得临床推广。

由于本实验为回顾性研究，无法做到随机化，所以实验中会存在偏倚。临床研究存在的偏倚大体上分为3种情况：选择性偏倚、信息偏倚以及混杂偏倚。本研究分别使用以下方法对其进行控制。

对于选择性偏倚，本研究搜集了全面的临床资料，来描绘患者的各项临床特征。其中，对于研究人群的选择，本研究为了排除其他基础疾病的影响，更好地对比两组患者的远期疗效，严格细化了两组的纳入、排除标准，同时排除了预期生存短、全身状况差的患者，选择全身状况好、没有基础疾病的患者作为研究对象。虽然本研究得出的生存期只能代表一般情况好、预后较好的部分晚期肺癌患者，但是，就本研究的研究目的而言，这样的研究对象的选择无疑更好的对比了两组的远期生存，尽可能降低了选择性偏倚对实验结果的干扰。另外，本研究还通过基线数据的对比来证实两组间的基线数据没有统计学上的差异，充分体现了两组间临床资料的可比性。

对于已知的混杂偏倚，本研究通过*Cox*比例风险模型来校正，可以很好的控制混杂因素对于实验结果的影响作用。

对于信息偏倚，本研究严格对随访员与数据录入员的培训，统一随访及录入标准，并且实现了数据的两次重复搜集，尽量降低人为因素导致的信息偏倚。

本研究在控制偏倚上做了以上工作，目的是为肺癌射频消融术提供一个相对较高的循证医学证据，是一个相对精确的观察性研究。与以往的研究相比，本研究的重点放在患者的生存期上，随访时间相对较长，患者终点事件的数目也较多，从一个观察性研究的角度，很好地提出了肺癌射频消融术联合化疗可以提高患者生存期的论点。但本研究依然存在一些弊端，如无法随机化、不能完全控制信息偏倚等，尤其是由于本研究观察终点为生存期，观察方式为电话随访，研究者无法亲自观测结局信息，而是通过患者家属的口头表达来确定结局事件，可能会导致信息偏倚的存在，但这也是观察性研究所共有的、无法避免的弊端。我们目前正在进行新一轮的随机对照试验，以无疾病进展期为研究终点来进一步论证肺癌射频消融术的远期疗效，以期为肺癌RFA提供更高级别的循证医学证据。

RFA作为一种优秀的微创手术方法，自2000年Dupuy教授首次应用于肺癌^[4]^以来，如今依然在蓬勃发展。尤其是随着射频电极等核心技术的进一步优化，RFA的适应证也渐渐拓宽，进入了越来越多学者的视野。但是，RFA的临床推广依然还有很多问题需要解决。首先，RFA对于各个适应证尤其是晚期肺癌的近期疗效与远期疗效，目前仍然存在争论，这无疑对RFA的临床推广形成了阻力。其次，RFA联合化疗对于疗效的协同作用，其机制目前仍然停留在假说阶段，并没有在基础研究的层面上被证实，如果上述的假说被证实，势必会使RFA的临床推广更加顺利。最后，由于新的治疗方法正不断出现并快速更新，对于减瘤手术而言，微创外科、RFA、立体定向放射治疗都是成熟而又具有竞争力的治疗方法，我们需要前瞻性的随机对照试验来对比这些减瘤手术之间的优劣，选择临床效果更优秀的方法进行推广。
